# Ultrasonographic Guidance and Characterization of Cryoanalgesic Lesions in Treating a Case of Refractory Sural Neuroma

**DOI:** 10.1155/2011/691478

**Published:** 2011-12-18

**Authors:** Ellen E. Rhame, Alexander F. DeBonet, Thomas T. Simopoulos

**Affiliations:** Interventional Pain Management, Arnold Pain Management Center, Harvard Medical School, Beth Israel Deaconess Medical Center, Brookline, MA 02445, USA

## Abstract

The recurrent pain of a neuroma following surgical excision and burial of nerve endings can be clinically challenging to manage. Cryoanalgesia in conjunction with ultrasound guidance was used successfully to manage this type of pain. Furthermore, ultrasound provided visualization of the cryolesions, as well as the relationships of the ice ball to the surrounding tissue. Following the completion of the freeze cycle, the tissue can be monitored for return to its usual morphology during the thaw period.

## 1. Introduction

Cryoanalgesia is a technique intended to provide pain relief in which low temperatures are used to generate soft tissue lesions [1]. It was first executed by Cooper et al. in 1962 with an early method that involved a hollow tube with an insulated tip, utilized liquid nitrogen as a coolant and achieved a temperature of –190°C [2]. Lloyd et al. first used the term “cryoanalgesia” for pain relief in 1976 [3]. They described a technique in which the exposure of painful tissue to locally subphysiologic temperatures could produce an effective reversible conduction block of peripheral nerves without the subsequent development of neuralgia. A major limitation in the characterization of a percutaneously generated cryolesion is that the lesion itself cannot be visualized in the closed application, and the only means of confirmation of appropriate probe function is by the use of a thermocouple to monitor temperature. However, use of the thermocouple does not provide details of ice ball formation. Modern ultrasound technique may allow this visual limitation to be overcome and provide greater ability to assess and even quantify the dimensions of a cryolesion in real time as it is being produced to provide a therapeutic endpoint during the cryoablation procedure.

Conventionally, pain physicians have used anatomical and fluoroscopic landmarks as well as sensory and motor stimulation to confirm the proximity of the cryoprobe to a target nerve. In a consideration of safety, this provides only a crude avoidance of potentially critical structures such as adjacent critical blood vessels, which may be avoided with ultrasonographic guidance. From a therapeutic standpoint, ultrasound allows imaging of many peripheral nerves and thus can provide visual confirmation that a cryolesion has encompassed the target nerve and enables spacial adjustments to be made as necessary. Ultimately, the employment of ultrasound guidance will likely increase the therapeutic success and reduce patient morbidity involved in cryotherapy.

 We describe a case of sural neuroma, in which ultrasound guidance was used to visualize in real time the lesion generated during a cryotherapy procedure. Although the use of ultrasound guidance in cryoanalgesia has been described, to the best of our knowledge the ultrasonographic image of a cryolesion itself for use in treatment in peripheral nerve cryotherapy has heretofore not been demonstrated in the pain medicine literature [4–6].

## 2. Case Report

A fifty-seven-year-old male with a past medical history significant for asthma and hypertension presented with a chief complaint of distal left lower extremity pain. At the age of twenty-one he was struck by a motor vehicle and sustained multiple fractures as well as a significant degloving injury to his distal left lower extremity. He underwent multiple extensive surgeries involving bone and skin grafts. Approximately six years prior to presentation at our clinic, he experienced a considerable exacerbation of his pain symptoms, was diagnosed with a sural neuroma, and eventually underwent surgical excision of the neuroma. The sural nerve endings were buried deep into the muscle just above the periosteum.

**Figure 1:**
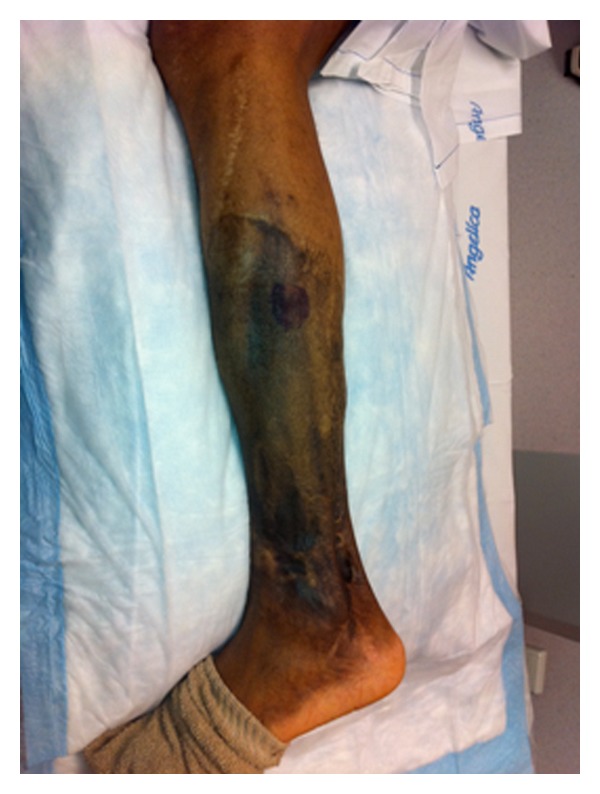
The blue skin marker demarcates the area of maximum pain in the hyperpigmented skin graft site of the left lower extremity.

Unfortunately, his pain returned which he described as burning, shooting, and located over his left lateral leg distal to the knee extending down to the ankle. The pain was exacerbated with palpation, eversion of the foot, and prolonged standing. He was treated with physical therapy, gabapentin, amitriptyline, and TENS unit with minimal benefit. At the time of presentation, he was taking desipramine 25 milligrams (mg) nightly and hydrocodone-acetaminophen 5 mg/500 mg as needed, which alleviated only a portion of his pain. On examination, he was a well-appearing man in no apparent distress. His left lower extremity distal to the knee revealed skin grafts, scarring, and hyperpigmentation (Figure 1). Hyperalgesia was present over the left lateral leg approximately halfway between the knee and ankle. This region had a positive Tinel's sign over a portion of the scar. There was no distinct palpable mass. The rest of the standard neurological examination was within normal limits. 

In March of 2007, the patient received an injection of the sural neuroma site with local anesthetic and steroid. He experienced significant relief for only the duration of the local anesthetic effect. He was then scheduled for cryoneurolysis of the sural neuroma. Throughout a period of 3 years in our clinic, he reported excellent relief from cryoneurolysis for 3 months following each serial treatment. In fact, he reports that for a period of approximately 10 weeks following the procedure, “I forget that I even have a pain in my leg.”

**Figure 2:**
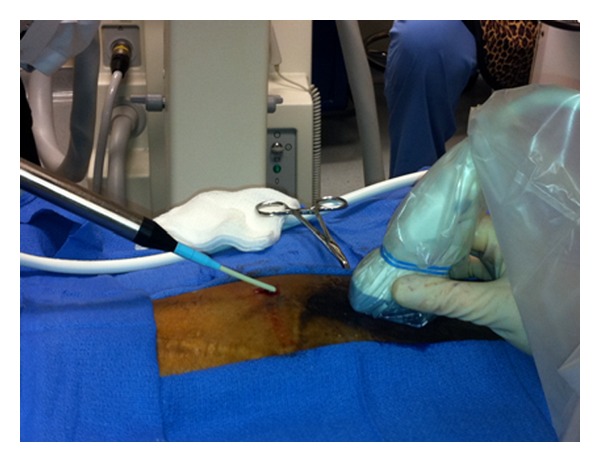
The cryoprobe was inserted via a 12-gauge angiocatheter introducer proximal to the skin graft site of the left lower extremity. The ultrasound probe is being utilized to provide an in-plane view of the cryoprobe and ice ball location during cryoneurolysis treatment.

Because of imaging limitations with fluoroscopy, ultrasound was used to better characterize the relationship of the cryoprobe in soft tissues relative to the fibula. The patient was placed in the right lateral decubitus position, and sterile prep and drape were performed using chlorhexidine/alcohol. Three milliliters (mL) of 1% lidocaine was infiltrated subcutaneously using a 27-gauge needle at the entry site for the cryoprobe located approximately 2 centimeters (cm) proximal to the skin grafts (Figure 2). Using an 11 blade, a 3-millimeter (mm) stab skin incision was made through which a 12-gauge angiocatherer was inserted into the subcutaneous tissue with the aid of ultrasound guidance (General Electric Venue 40 ultrasound system and 12 L linear probe). Ultrasonographic examination of the corresponding painful site did not allow visualization of an apparent sural neuroma. A 14-gauge cryoprobe (Wallach WA5000 Painblocker) was inserted through the 12-gauge angiocatheter upon removal of the needle. Proper sensory stimulation was used to confirm proper placement of the cryoprobe at 125 Hertz (Hz) to reproduce paresthesias over the patient's typical location of pain. The cryotherapy session consisted of 6 individual cryolesions, each of 4 minute duration, with a 40-second thaw period between each lesion.

**Figure 3:**
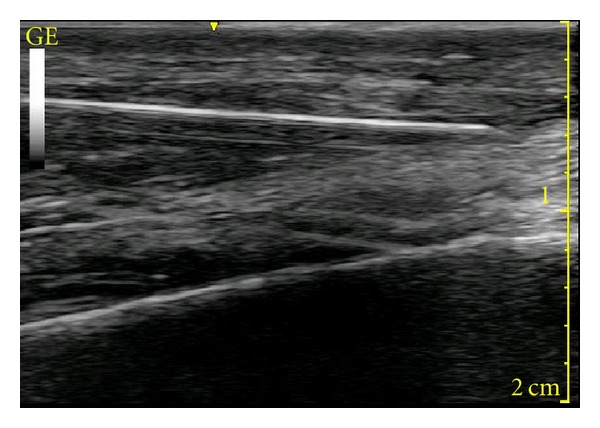
The in-plane view of the cryoprobe and fibula prior to cryolesioning.

**Figure 4:**
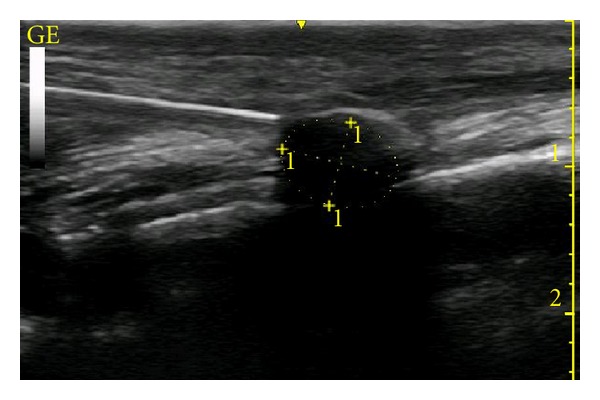
Cryolesion (diameter = 0.80 cm) at 1 minute and 30 seconds into the freeze cycle. Note the hyperechoic rim with posterior acoustic shadowing emanating from the tip of the probe.

**Figure 5:**
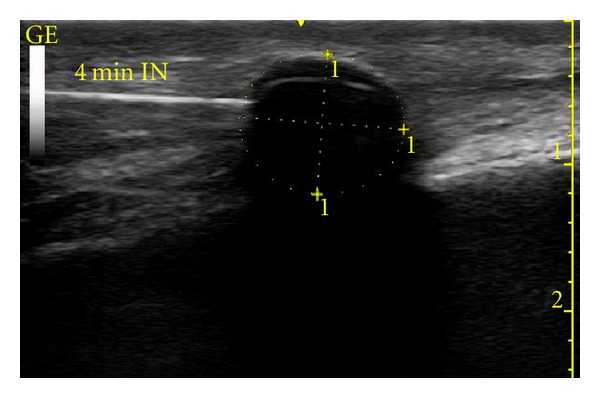
Cryolesion (diameter = 1.12 cm) at 4 minutes into the freeze cycle.

**Figure 6:**
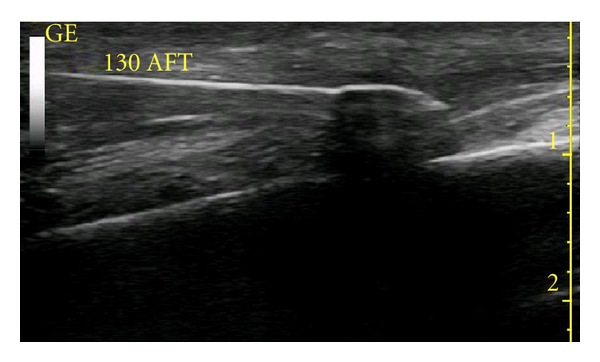
Cryolesion is barely visible at 1 minute and 30 seconds into the thaw period.

During the beginning of the procedure, the cryolesion and cryoprobe were imaged using ultrasound both in-plane and out-of-plane view. The proximity of the cryoprobe to the fibula was easily visualized prior to beginning cryotherapy (Figure 3). Any apparent vascular structures were readily avoided during cryoprobe insertion under direct in-plane ultrasonographic guidance and doppler examination. The spacial magnitude of the cryolesion relative to the cryoprobe was easily visualized under real-time ultrasound. The lesion appeared as an echo-opaque hemisphere with a hyperechoic rim with a posterior acoustic shadowing (Figure 4). The lesion was easily appreciated as increasing in size radially throughout the 4-minute period as well as decreasing radially during the 40-second thaw period between lesions. The diameters (in cm) of the lesions were 0.80, 0.87, 1.01, and 1.12 at 1.5, 2, 3, and 4 minutes, respectively. The short axis view confirmed the spherical nature of the lesion at the tip. The largest ultrasonographically measured cryolesion was an area of 0.85 cm^2^ and a circumference of 3.28 cm, at 4 minutes into freezing cycle (Figure 5). It must be taken into consideration that because of the posterior acoustic shadowing cast by the cryolesion, as its radius increases the dimensions of the posterior portion of the cryolesion must be approximated for measurement purposes. The cryoprobe was positioned so that the posterior portion of the cryolesion would include the periosteum of the lateral mid-fibula where the symptomatic neuroma was believed to lay. After a 4-minute cryolesion, the tissue changes were visualized at 50 seconds but then normalized at 2 minutes and 20 seconds (Figure 6). The patient tolerated the procedure well without apparent complications. Sequential freezing and thawing pictures as presented in Figures 3–6.

## 3. Discussion

Increased utilization of ultrasound is becoming more commonplace in both acute and chronic pain therapies. Aside from offering image guidance, other potential benefits of ultrasound include decreased radiation exposure, increased portability, and decreased costs [5]. In this case of recurrent pain after surgical excision of a neuroma, ultrasound allowed identification of the ice ball in relation to the deep muscle, fascia, and periosteum. Ultrasonographic imaging has been shown to be an effective means of monitoring ice ball lesions during cryodestruction of liver tumors in the surgical literature [7]. It follows that the characteristics of an in vivo soft tissue cryolesion in the extremities can be determined as well.

The cryolesions of the liver and peripheral soft tissues, in this case mostly muscle/fascia, exhibited a similar morphology. The hyperechoic rim of the cryolesion has been described as a “freeze front” which advances during the freezing cycle and regresses during the thawing cycle [7]. The surgical literature also provides evidence that the ultrasound images of cryolesion and the gross macroscopic hepatic cryolesion correlate closely in actual size [8]. Previously, the ultrasonographic cryolesion has been described as a “hemispherical hyperechoic rim with posterior acoustic shadowing”, and this image was readily observed in this case study [9]. The hyperechoic rim is thought to be a result of increased acoustic impedence at the border between frozen and unfrozen tissues, which causes ultrasound waves to be reflected back towards the transducer [8]. The posterior acoustic shadow results in part from the decreased portion of ultrasound waves that is capable of passing though the frozen cryolesion. Following complete thawing of the cryolesion, no ultrasonographic differences were appreciated between tissues that had undergone cryotherapy and those that had not. This differs from cryotherapy of the liver, where the liver tissue that had undergone cryotherapy for 22 minutes at –180°C remained hypoechoic compared to surrounding tissues after complete thawing [7, 8].

Several factors contribute to the dimensions of a lesion generated during cryotherapy. The overall volume of the cryolesion itself is a function of the duration of the lesioning application and the temperature achieved during lesioning that may be hampered by the constant delivery of body heat by blood vessels or cerebrospinal fluid [10]. In clinical practice, the upper limit freeze duration is empirically set at 4 minutes, because little benefit is thought to be derived from longer durations [11]. The present images do suggest the majority of the lesion is established during the first two minutes, but continue to expand an additional 2.5 mm at the end of 4 minutes. On the other hand, repeated freeze-and-thaw cycles have been demonstrated to be associated with increased volume of the cryolesion [12]. Repeat freeze/thaw lesions were not evaluated in this report. Moreover, the therapeutic efficacy of the treatment improves as the placement of the cryoprobe targets more closely the peripheral nerve [10]. This allows for a greater length of a peripheral nerve to be lesioned, thereby impairing saltatory conduction so as to prevent pain transmission [13]. In cats it has been determined that a cryolesion of 3–6 mm in diameter was necessary to prevent saltatory conduction [13]. While this agrees with prior clinical reports [14], the estimations in this study are nearly twice as large in diameter. This disparity may be based on the size of the probe, differences in cryodevices, and the surrounding tissue milieu. In any event, a larger lesion would be preferable for ensuring an ice ball has interacted with sufficient length of the target nerve. This feature is particularly useful when target nerves are not readily visualized as in this case.

In usual clinical practice, an adequate thawing interval following a cryolesion is desirable. This is because if the cryoprobe is moved before complete thawing has occurred, damage may transpire to surrounding tissues that remain adherent to the cryoprobe while frozen. A time period ranging from 20th–40 seconds for a thaw interval prior to repositioning the cryoprobe before starting the next freeze cycle has been used clinically for years without reported adverse events [10]. After a 4-minute cryolesion, tissue changes were very apparent at 50 seconds and did not normalize until 140 seconds later. Though ultrasonographic data would suggest a 140-second thaw interval after a 4-minute cryolesion, there have been no adverse reports using the shorter aforementioned intervals. Therefore it is difficult to recommend increasing the time for thawing, but warrants further investigation.

## 4. Conclusions

Ultrasound techniques offer valuable guidance of cryoprobes into deep soft tissue that is in close proximity to periosteum where transposed nerve endings lie. Furthermore, ultrasound can be useful in characterizing the dimensions of ultrasound lesions and their relationships to surrounding tissues. Lastly, this imaging modality can also be used to determine when the tissue following a cryolesion returns to its usual morphology.
